# Headache Attributed to Reversible Cerebral Vasoconstriction Syndrome (RCVS)

**DOI:** 10.3390/diagnostics13172730

**Published:** 2023-08-22

**Authors:** Vasileios Tentolouris-Piperas, Loukas Lymperopoulos, Argyro Tountopoulou, Sophia Vassilopoulou, Dimos D. Mitsikostas

**Affiliations:** 1st Department of Neurology, Eginition Hospital, School of Medicine, National and Kapodistrian University of Athens, 11528 Athens, Greece; vastentolouris@gmail.com (V.T.-P.); louklymper@gmail.com (L.L.); atounto@yahoo.gr (A.T.); svasilop@med.uoa.gr (S.V.)

**Keywords:** reversible vasoconstriction syndrome, RCVS, thunderclap headache, headache, cerebrovascular disease

## Abstract

Reversible cerebral vasoconstriction syndrome (RCVS) is a condition with variable outcomes presenting a new onset thunderclap headache accompanied by focal neurological symptoms or seizures. It can be idiopathic or arise secondarily to a variety of trigger factors. The condition is increasingly recognized in clinical practice, but many facets remain poorly understood. This article aims to clarify the headache characteristics in RCVS, the temporal association of angiographic findings, the potential association of the condition with SARS-CoV-2 infection, and the clinical presentation of RCVS in children and is based on a systematic PRISMA search for published analytical or large descriptive observational studies. Data from 60 studies that fulfilled specific criteria were reviewed. Most people with RCVS exhibit a typical thunderclap, explosive, or pulsatile/throbbing headache, or a similar acute and severe headache that takes longer than 1 min to peak. Atypical presentations or absence of headaches are also reported and may be an underrecognized phenotype. In many cases, headaches may persist after resolution of RCVS. Focal deficits or seizures are attributed to associated complications including transient ischemic attacks, posterior reversible encephalopathy syndrome, ischemic stroke, cerebral edema, and intracranial hemorrhage. The peak of vasoconstriction occurs usually within two weeks after clinical onset, possibly following a pattern of centripetal propagation, and tends to resolve completely within 3 months, well after symptoms have subsided. There are a few reports of RCVS occurring in relation to SARS-CoV-2 infection, but potential underlying pathophysiologic mechanisms and etiological associations have not been confirmed. RCVS occurs in children most often in the context of an underlying disease. Overall, the available data in the literature are scattered, and large-scale prospective studies and international collaborations are needed to further characterize the clinical presentation of RCVS.

## 1. Introduction

Reversible cerebral vasoconstriction syndrome (RCVS) is a self-limiting condition attributed to transient cerebral vasospasm, presenting with a severe headache and/or focal neurological symptoms. As a presenting symptom, a headache usually has thunderclap characteristics. It recurs for about 1–2 weeks and it may precede an ischemic or hemorrhagic stroke. RCVS may also occur in the context of several predisposing factors, including vasoactive substances, blood products, and the postpartum state [1]. The term reversible cerebral vasoconstriction was first mentioned by Call and Flemming in 1988, who reported four cases of abrupt headache and reversible cerebral vasoconstriction. Combined with 12 more cases of patients with similar clinical characteristics, they hypothesized a new clinical entity initially termed “Call–Flemming syndrome” [2]. Following that, case reports of patients with common characteristics of headache and reversible cerebral vasoconstriction were published using various terms such as benign angiopathy of the central nervous system (CNS), postpartum angiopathy, cerebral vasculopathy, or isolated benign cerebral vasculitis. The term reversible cerebral vasoconstriction syndrome has been widely adopted after the publication of two seminal articles in 2007, reviewing and unifying the previous terminology and presenting the first large series of patients [3,4]. The proposed key diagnostic criteria have since been slightly modified [5].

RCVS is a rare syndrome with a mean age of onset in the fifth decade of life, showing female predominance in adults at a ratio ranging from 2:1 to 10:1 [3,6,7,8]. Formal epidemiological data are lacking, but incidence has been estimated to be 0.3/100.000 annually in the United States [9]. The true incidence is hypothesized to be higher, and in recent years, our understanding of the clinical course and imaging findings of RCVS has improved, making this entity more easily recognized and diagnosed. However, it can still manifest in a variety of clinical scenarios, which, in combination with its clinical and imaging heterogeneity, often makes definite diagnosis a challenge, particularly in the acute setting. Initial diagnosis requires radiographic proof of vasoconstriction, which frustratingly may not be apparent early in the disease course. Even if it is, it could be missed with the imaging modalities commonly available in an emergency department. Finally, reversibility of vasoconstriction within 3 months of symptom onset is required for definitive diagnosis, but this is by definition impossible to know early on. However, time is of the essence, as RCVS is known to harbor dangerous complications such as hemorrhage, ischemic stroke, cerebral edema, and seizures. Early diagnosis and differentiation from other possible diagnoses is imperative for appropriate management.

Headache attributed to reversible cerebral vasoconstriction syndrome (HaRCVS) is a broad classification introduced in the latest International Classification of Headaches (ICHD-3 6.7.3.1) that describes new-onset headaches in patients with confirmed RCVS, which completely resolve within 3 months of onset and fulfill one or both of the following criteria [10]:

1 The appearance of the headaches themselves led to the diagnosis of RCVS and/or;
2 Headaches possess at least one of the following characteristics:
  (i) Thunderclap onset;
  (ii) They are triggered by sexual activity, exertion, Valsalva maneuvers, emotion, and bathing/showering;
  (iii) They are present or recurrent for at most one month after onset and then subside.

In the absence of angiographical confirmation of RCVS, the criteria that allow for the diagnosis of probable HaRCVS (6.7.3.2) (hence, probable RCVS) are much stricter, and are naturally closer to what is generally known as the clinical presentation of RCVS. They require the headache to be severe and have a thunderclap onset, peaking within one minute and lasting at least 5 min, while having been triggered by at least one of the aforementioned trigger factors. The criteria further allow for persistent headaches associated with past RCVS to be diagnosed if the symptoms persist for more than 3 months (6.7.3.3). To reach a higher clinical suspicion for RCVS as early as possible, differentiate it from other conditions as accurately as possible, and inform further diagnostic and therapeutic decisions, two diagnostic scores have been devised, RCVS2 [11] and RCVS-TCH [12]. The RCVS2 score greatly incorporates the presence of thunderclap headache (TCH) in the prediction model and RCVS-TCH considers TCH as a prerequisite. However, there are many RCVS patients presenting an acute, severe headache that does not strictly adhere to the TCH definition, as well as patients with different headache presentations or no headache at all.

The aim of this review is to present the key features of HaRCVS and accompanying focal symptoms in relation to the imaging findings with various modalities via a systematic review of the published analytical observational studies (large case series, cohort, or case–control studies).

## 2. Materials and Methods

**Figure 1:**
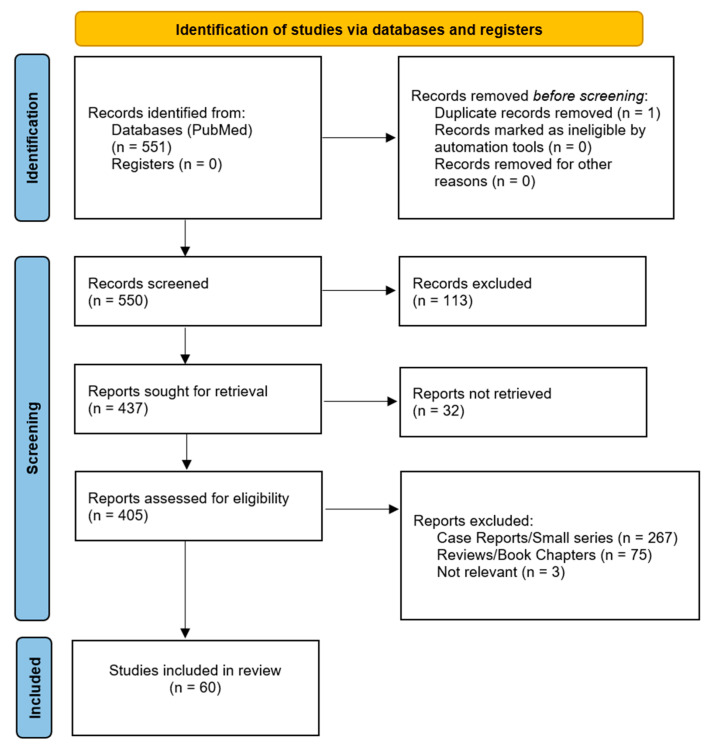
Flowchart depicting the process of the literature search (PRISMA chart).

The review of the literature was conducted following the Preferred Reporting Items for Systematic Reviews and Meta-Analyses (PRISMA) guidelines [13]. Our search was conducted in PubMed using the search terms (reversible cerebral vasoconstriction) AND (headache) on 17 April 2023 and was repeated on 27 June 2023 to add any latest publications. No date or language restrictions were applied. Two separate members of the writing team (L.L. and V.T.-P.) reviewed the literature independently and conducted the filtering. Initial review of the titles and abstracts excluded correspondence and editorials, case reports, duplicates, and articles that were clearly irrelevant, or whose full text was in a language other than English. A second round of reviewing the full text excluded small descriptive studies (case reports and small case series of fewer than 10 patients), reviews, and other studies that were not relevant to the topic. The end result of this search was large descriptive studies (with a total cohort of at least 10 patients), analytical observational studies, and trials. The final results of this search were then read in detail to extract relevant information for the headache characteristics of RCVS patients. Articles excluded in the 2nd round were also read and incorporated in the discussion section if they contained relevant information or insights into the topic of this review. Additional unfiltered literature searches were performed for children and COVID-19. A flowchart of the literature search process and the results is shown in Figure 1.

## 3. Results

### 3.1. Results of the Literature Search

No published clinical trials pertaining to RCVS patients were found. Sixty observational studies fulfilled our criteria with implications on RCVS [3,6,7,8,11,12,14,15,16,17,18,19,20,21,22,23,24,25,26,27,28,29,30,31,32,33,34,35,36,37,38,39,40,41,42,43,44,45,46,47,48,49,50,51,52,53,54,55,56,57,58,59,60,61,62,63,64,65,66,67]. Of those, 16 studies contained sufficient information on particular headache characteristics of their cohorts, and they are presented and summarized in Table 1.

Results not included in Table 1 focused on postpartum patients, vascular events, white matter abnormalities, imaging, or other conditions in the differential for RCVS that did not contain enough information on headache characteristics of their RCVS cohorts to be included in the table. However, they concern other important facets of the syndrome and are further discussed in the text, where appropriate.

### 3.2. Headache Associated with RCVS

#### 3.2.1. Typical Thunderclap Headache and Thunderclap-like Headache

Thunderclap headache has long been considered the hallmark symptom of RCVS since the syndrome was first described. The hallmark case series, which was published in 2007, reported a 98.5% presence of TCH headaches in RCVS patients [3] using the ICHD-2 TCH criteria [68].

Some studies in our search used the presence of thunderclap headache as a prerequisite to identify and recruit patients [6,21,37,60] and, therefore, all patients had TCH, although various definitions have been used. Studies that opted for different methods to identify RCVS patients recruited cohorts of patients with a wider variety of presentations, leading to a lower reported incidence of TCH in RCVS patients, ranging from 67 to 86.5% [7,18,25,42].

In some reports, TCH is not strictly defined [7,37,52], while in others, the definition is inadequate or is not reported at all [28,33,59,61,67]. In studies that strictly used the TCH definition, most of the non-TCH patients experienced headaches with similar characteristics: severe intensity and fast onset-to-peak, but not as fast as 1 min [3,18,25,42]. In studies not reporting the TCH definition used, most patients are still reported to have TCH or similar acute and severe headaches [7,52,61,67].

The character of the pain is usually described as explosive or pulsatile/throbbing, but it may also be dull or tearing [37,47]. Intensity tends to reach more than 7/10 on the numeric scale by definition of TCH, although many studies do not report quantitative evaluation. Smith et al. report patients who have “thunderclap” headaches of as low as 4/10 on the intensity scale [60]. Furthermore, headache intensity tends to diminish from episode to episode until full remission [7].

Location of pain has not been found to be particularly helpful in distinguishing RCVS and has not been adequately reported in all studies. For example, initial information that pain starts occipitally and then quickly spreads diffusely was reported in an early case series [3] and has since been cited in the majority of subsequent studies and reviews. However, not many corroborating data have been published since, and newer publications with larger cohorts do not report such information at all. The exception to this observation is a cohort of 174 RCVS cases triggered by sexual activity: 67% of patients had bilateral pain, with the most common locations being occipital (69%) and temporal (50.6%), but all locations, as well as diffuse pain, were reported. Pain is most commonly bilateral, but a unilateral location may be present in 20–33% of patients [3,21,47].

Headaches in RCVS patients tend to happen episodically [3,23,47], but patients with a single episode were also initially described [3]. A monophasic course may be more common, as much as 40% [18]. Episodic headaches within the first weeks are a sign that strongly suggests a diagnosis of RCVS compared to other causes of TCH [69].

The duration of each attack usually lasts for 3–5 h [3,21] and can range from several minutes to several hours or even a day and more [3,23,37,47]. A duration of less than one hour was less common in RCVS patients compared to other causes of sudden headaches [23]. The total duration of the disease until headaches remiss completely is around 2–3 weeks [3,7], but can range from 2 days to 4 months [37].

It is important to note that some patients may have thunderclap at onset, and others later in the disease course, or even never. A variety of headache types can occur in the same period of disease [42].

#### 3.2.2. Presentation with Atypical Headache or No Headache

As discussed, RCVS can present without the typical thunderclap headache. Most commonly, this still refers to acute onset, severe headaches that peak quickly in more than 1 min; hence, this presentation was discussed above. However, there are also cases of RCVS presenting with severe but much more gradual headaches, non-severe headaches, or no headaches at all. In later publications, atypical presentations are being discussed more often, ever since a review of published case reports nicely elucidated this subset of RCVS patients [70]. In a retrospective study aiming to characterize TCH in RCVS patients, nine patients from the initial RCVS cohort were excluded from the analysis due to presenting atypical headaches. Unfortunately, no further details were reported [60]. This example highlights the bias that exists in the current literature and that atypical presentations may generally be even more prevalent. In the cohorts identified by our search, examples of other headaches include severe headaches peaking up to 120 min from onset, unusual progressive headaches, and headaches appearing upon waking with varying intensity [18,25,42]. No matter the characteristics, patients still describe these headaches as unusual.

There are also reports of patients who do not have any headaches, which are also summarized by Wolff et al. [70]. Our search provided published studies where such cases were also part of cohorts [7,23,25,33,42,59], although not much information could be gleaned about the details of the clinical presentation in this particular subgroup. Commonly, these patients are usually discovered through complications of the syndrome, such as ischemic stroke or hemorrhage. Since headache is still considered the hallmark of RCVS, cases without headaches could be missed. It is also plausible that RCVS can occur subclinically or produce such minor and insignificant symptomatology that patients never seek care for and are never evaluated.

#### 3.2.3. Accompanying Symptoms of Headaches

The symptoms accompanying headaches of RCVS tend to resemble those of SAH and meningeal irritation; however, they have not been adequately elucidated. One reason is that RCVS can occur with transient or permanent deficits and seizures, and it is not always clear if part of the presentation is due to the headache or the complications. From our search, Caria et al. reported nausea and vomiting in 81.2% of RCVS patients with TCH [18], while Lin et al. reported nausea, vomiting, photophobia, and phonophobia in patients with RCVS that presented a headache after sexual activity [47]. Most of these patients did not have SAH, so symptoms can be more easily attributed to RCVS and the headache itself. Kim et al. reported nausea in seven of eighteen patients with RCVS and TCH, as well as rarer cases of ocular pain, vomiting, dizziness, and phonophobia. Generally, pain is often accompanied by screaming and crying (32%), photophobia (30%), nausea (57%), and vomiting (38%) [3]. Phonophobia has also been reported elsewhere [6,37,47]. Extremely intense headaches can often lead to sympathetic overactivity; therefore, crying, agitation, panic, screaming, fear of dying, confusion, or collapse are common accompanying symptoms [71]. Furthermore, about one-third of patients have a surge of blood pressure (systolic blood pressure > 160 mmHg) accompanying headache attacks [69,72]. This may be a response to the excruciating pain but may also be part of the underlying processes or vascular complications. Nevertheless, as a finding, it is an important clue to the presence of RCVS, and it has been incorporated into diagnostic scores [12]. It is not clear if any of these accompanying symptoms tend to occur in non-TCH RCVS patients as well.

#### 3.2.4. Persistent Baseline Headaches

Although RCVS headaches typically occur in episodes, some patients experience the presence of a persistent headache between episodes. The presence of such a headache should not discourage the diagnosis of RCVS if the rest of the presentation is compatible. According to the pivotal observational study of the Ducros group, a headache of lower intensity persisted between thunderclap attacks in approximately 35% of cases [3]. As seen in Table 1, similar findings were reported in the Italian cohort [18], in cohorts of RCVS patients with TCH [21,60], and in cohorts of RCVS patients presenting sudden headaches [23], and acute ischemic stroke [67]. However, all noteworthy headaches in the vast majority of cases should be gone by 3 weeks after onset [5].

#### 3.2.5. Trigger Factors

There is a wide variety of trigger factors that have been described in association with RCVS in the literature. The term “secondary RCVS” has been adopted by some authors for cases with a confirmed, identifiable trigger. In general, these mainly include emotional, postural, or environmental triggers, the intake of vasoactive substances, medical conditions, and pregnancy or recent childbirth. Some of these triggers have been well-described and their correlation with RCVS is undoubtful, while others seem more dubious, as they involve common, everyday situations or have a substantial delay between exposure and development of the syndrome [73]. TCH may start upon first exposure to an associated substance, or after long-term use at normal or excessive doses [71].

Commonly described precipitating factors include Valsalva maneuvers, coughing, sexual activity, physical exertion, contact with water, urinating without effort, sneezing, laughing, singing, emotional triggers (mostly negative), and sudden forward flexion [3,23,25,42].

The postpartum period has also been strongly associated with RCVS. A study by Lange et al. on 345 patients found that recent childbirth was a precipitating factor in 8% of patients [42]. On the other hand, RCVS seems to be rare among postpartum individuals, as shown in another prospective study, where out of 900 postpartum women, 34 developed TCH, but only 1 was diagnosed with RCVS [15]. According to Singhal et al., it usually emerges within1 to 3 weeks following an uncomplicated pregnancy, although presentation can occur as late as 6 weeks after delivery [74]. More recent studies have shown that RCVS emerges in a shorter time span, specifically 1–10 days after delivery, according to Topcuoglu et al. [64]. For this reason, RCVS was previously known as postpartum angiopathy [75]. Almost half of the patients have a history of proteinuria, suggesting overlap with pre-eclampsia, and there is also an association with HELLP syndrome (hemolysis, elevated liver enzymes, and low platelets), as well as posterior reversible encephalopathy syndrome (PRES) [76].

Vasoactive substances such as illicit drugs, α-sympathomimetics, and serotoninergic drugs are considered among the most common precipitating factors [42,53,77]. Drugs that may induce RCVS include selective serotonin reuptake inhibitors (SSRI) (such as escitalopram, paroxetine, sertraline) and triptans [18]. Other medications, such as nasal decongestants, have also been reported [3]. A very interesting case was described by Abruzzo et al. involving a 19-month-old female patient with retinoblastoma, who underwent selective ophthalmic artery infusion chemotherapy. Shortly after administration of a series of substances including intranasal oxymetazoline, she developed systemic hypertension and cerebral vasoconstriction, which spontaneously resolved within hours without any further complications [78]. Regarding illicit drugs, cannabis is the only one that has been associated with the development of RCVS. There is a lack of evidence for other illicit drugs (cocaine, ecstasy, amphetamine, methamphetamine, khat, heroin, LSD) as single precipitating factors [57,79].

Other reported triggers include alcohol, blood products, head trauma, neurosurgical procedures, headache disorders (exertional headache, migraine, primary thunderclap headache, benign sexual headache), tumors (in particular, pheochromocytoma and paraganglioma), vascular conditions and procedures (fibromuscular dysplasia, endovascular procedures, cervical artery dissection, unruptured intracranial aneurysm, carotid endarterectomy), and antiphospholipid syndrome [80,81,82]. RCVS in association with thrombotic thrombocytopenic purpura has also been reported [83]. Trauma/surgery/procedure as precipitating factors have been linked to poor clinical outcomes [77].

Overall, the percentage of patients with identifiable trigger factors can significantly vary between studies, as some studies suggest a lower percentage (55.8%), while others suggest a percentage up to 80–90.1% [3,6,42]. The prospective cohort study by Choi et al. identified trigger factors in only 16/104 patients with definite RCVS, with the most common being recent childbirth (5.8%) and medication use (5.8%), while 33/104 patients reported a preceding event or change in lifestyle, environment, health, or medication within one month before onset, which could not be definitively identified as a trigger [25]. In contrast, a recent review of 191 cases reported identifiable trigger factors in 88.5% of patients [77]. This extreme variability could be attributed to different trigger factors taken into account in each study or a publication bias of cases with precipitating factors, which may not represent real-world data. Secondary RCVS has been associated with atypical clinical manifestations and a higher rate of complications [18].

#### 3.2.6. Persistent HaRCVS and Recurrency

It seems that RCVS as a phenomenon is not strictly monophasic, although most patients tend to experience it only once. In a cohort study, 6/77 patients (8%) had a recurrence during a median follow-up of 25 months (range 10–69) [6]. In a follow-up study, the same group of researchers demonstrated a recurrence of TCH in 18 of 169 RCVS patients with a long-term follow-up (mean of 37.5 months). Vasoconstriction was re-demonstrated in nine of these patients (5.4%) [22]. Another prospective study showed that 10 of 172 patients (5.6%) were found to have a recurrent episode of RCVS, though none of these recurrent cases resulted in permanent sequelae [17]. One patient in the same study had a third recurrence of RCVS. Predicting factors that were found to be associated with the recurrence of RCVS included a history of migraine and having exercise or sexual activity as a trigger for thunderclap headaches during the initial episode of RCVS [17].

There are studies suggesting that RCVS not only causes excruciating headaches in its acute phase, but also leads to chronic headaches afterward [36,84]. Rozen et al., in 2013, first reported a patient with possible RCVS (although not diagnosed as such in the study), followed by new daily persistent headaches. The authors considered this presentation as a novel phenotype [85]. Jamali et al. described three cases of RCVS followed by new daily persistent headaches [86]. A retrospective two-centered study, conducted by the Cleveland Clinic Foundation and Massachusetts General Hospital in 2016, investigated the long-term outcomes of RCVS patients. The study provided important information regarding headaches after RCVS resolution. Around half (24/45) of the patients continued to have chronic headaches after RCVS resolution [36]. In a recent study of long-term effects, 50% of patients were found to experience long-term headaches, defined as headaches beyond 6 months from onset [39]. This chronic headache, which can be triggered by RCVS, has lately begun to be recognized and described even further. It is now formally included in the third edition of the International Classification of Headache Disorders (ICHD-3, 2018) and is named “persistent headache attributed to past RCVS” [10]. Psychological distress and a prior history of migraines are regarded as predisposing factors for the occurrence of post-RCVS headaches [87].

#### 3.2.7. Focal Neurologic Deficits, Seizures, Strokes, and Other Accompanying Conditions

Even though the majority of patients (up to 75%) describe headaches as their only symptom, fluctuating neurologic deficits and seizures are also commonly described [88]. According to various studies, focal neurologic deficits may have an estimated prevalence between 8% and 63% [69,72,89]. According to Song et al., the most common neurologic symptom of RCVS is headache (93.2%), followed by motor weakness (36.6%), visual abnormality (30.4%), mental change (20.4%), and seizures (17.3%) [77]. In addition to the aforementioned symptoms, RCVS may present encephalopathy, dysarthria, aphasia, or ataxia [89]. Patients with aphasia/neglect/apraxia as acute neurologic symptoms had a poor clinical outcome with persistent neurologic deficits [77]. There might also be an association between the severity of RCVS and atypical clinical features since these patients can often present with neurological deficits due to stroke, severe PRES, seizures, confusion, or even coma [89,90].

The focal deficits are usually a consequence of associated complications, such as transient ischemic attacks (TIAs) (up to 16%), PRES (9–14%), ischemic stroke (4–54%), and intracranial hemorrhage (34–43%), specifically cortical subarachnoid hemorrhage (cSAH) (up to 22%) or intracerebral hemorrhage (ICH) (up to 6%) [29,65,69]. In the largest RCVS series published to date [32] of 1157 patients, 18.6% had AIS, 39.5% had a hemorrhagic stroke, 11.2% had cerebral edema, and 4.3% had a new-onset seizure. This sample of patients does not include patients who died in their first admission, only patients who were discharged, so rates of complications may be even higher among the entire RCVS population. However, patients were identified using discharge diagnosis codes and not diagnostic criteria.

According to a recent review, the incidence of hemorrhagic strokes and PRES is even higher (in 44% and 38% of cases with RCVS respectively) [72]. Specifically, an observational study performed by Patel et al. showed that the total frequency of hemorrhage in patients with RCVS was 43.4%, while the frequency of SAH, ICH, or subdural hematoma was 35.9%, 13.1%, and 3.6%, respectively [53]. There can be an overlap of different types of hemorrhage in the same patient. As far as patients with SAH are concerned, 90% present with it initially [80]. Intracranial hemorrhage tends to be small (less than 10 mm^3^) and usually precedes SAH, although they are both early complications [80]. Both types of hemorrhage (cSAH and ICH) and PRES are early events occurring usually during the first week, while ischemic events, including TIAs and cerebral infarcts, occur later, usually during the second week, and on average 8 days later than the hemorrhagic complications [3,90]. Ischemic strokes usually involve a watershed distribution between the anterior and posterior circulation, whereas major arterial territorial infarcts are uncommon [90]. Ischemic strokes are usually bilateral. Cerebellar strokes have also been described in RCVS, although they are rare [80].

Cerebral edema is a possible complication of RCVS and it has been reported in almost 10% of cases [91]. It usually occurs in the first week after clinical onset, showing complete resolution within 1 month [80].

Dissection of the cervical arteries may also be associated with RCVS, but it is unclear whether it is a cause or a downstream effect of the syndrome [73]. A cohort study of 20 patients has demonstrated cervical artery dissection in 12% of patients with RCVS, most frequently females with a migraine history and neck pain [50]. Interestingly, a retrospective study comparing RCVS patients with the control groups of primary angiitis of CNS (PACNS) and patients with other neurological syndromes who underwent angiographic evaluations indicated that patients with RCVS were more likely to have coexisting vascular abnormalities such as cervical artery dissection (CAD), aneurysms, and cavernomas [63]. Similarly, postpartum patients with CAD were more likely to have RCVS compared to non-postpartum patients [92].

#### 3.2.8. Diagnosis of HaRCVS and RCVS

Diagnosis of HaRCVS is simple but requires a prior confirmed diagnosis of RCVS; hence, it is not particularly useful in the acute setting. Strictly applying the ICHD-3 classification criteria means that any new type of headache in a patient that leads to a diagnosis of RCVS, regardless of the headache characteristics and if it occurs in isolation or not, is automatically termed as HaRCVS if it cannot be attributed to other causes. On the other hand, if RCVS is diagnosed in spite of the presence of a headache, any new persistent or recurring headaches that are present afterward, subside within one month, and are not otherwise explained, can also be termed as HaRCVS.

Diagnosis of RCVS itself is trickier because it requires proof of vasoconstriction and its reversal, something that occurs usually at least 1 month from disease onset. Aneurysmal subarachnoid hemorrhage should always be ruled out at symptom onset, along with vasoconstriction due to inflammatory conditions and all other potential causes of new-onset acute headaches. Imaging diagnostics will be examined in the following section. Lumbar puncture (LP) is of crucial importance for the diagnosis of RCVS in order to differentiate it from inflammatory diseases (such as PACNS) or SAH. In the majority of patients with RCVS, LP shows normal or near-normal results. Caria et al. found a slightly elevated cerebrospinal fluid (CSF) leukocyte count and slightly elevated protein level in a minority of patients [18]. CSF findings are more often normal in patients with RCVS compared to patients with PACNS [28]. In contrast to other studies, a small cohort showed that 5/6 of RCVS patients had leucocyte levels of more than 10/mm^3^ [38].

So far, three ways exist to suspect and diagnose RCVS early, before conducting extensive neuroimaging. These are the “Probable HaRCVS” criteria (6.7.3.2) of the ICHD-3, and the two diagnostic scores, RCVS2 and RCVS-TCH. The latter two have a primary goal of differentiating RCVS from other causes of vasoconstriction and thunderclap headaches, respectively. In a validation study, the criteria for probable HaRCVS (6.7.3.2) [45] were found to be specific for identifying RCVS in patients presenting with thunderclap headaches. The RCVS2 score and diagnostic approach can be used to promptly diagnose and distinguish RCVS from mimics, using easily available admission variables (the presence of thunderclap headache, the absence of intracranial carotid artery involvement, the presence of a vasoconstrictive trigger, gender, and the presence of subarachnoid hemorrhage) [11]. Kumar et al. supported the usefulness of the RCVS2 score in a real-world clinical context using a cohort of 55 patients and demonstrated that the RCVS2 score had a significant association with high specificity and positive predictive value for angiographic vasoconstriction [39]. Cho et al., however, developed a new prediction model, the RCVS–TCH score, which showed high specificity and sensitivity for discriminating RCVS in patients with TCH. The authors suggest that the RCVS2 score has an excellent performance to predict RCVS in patients with arteriopathies, but the RCVS-TCH score better helps distinguish RCVS from primary TCH or other secondary causes of TCH [12].

All three of these options have TCH in a central role. With recent publications suggesting a higher than previously thought prevalence of non-TCH presentations, it is not easy to suspect RCVS in the first place and request necessary diagnostic procedures. Future studies may shed light on this subgroup of patients and determine if there are definitive clues that may point to RCVS in this case. Nevertheless, new headaches or vascular events that cannot be explained otherwise, particularly in patients that report one of the known possible triggers, should evoke suspicion, even in the absence of TCH. A well-designed prospective case–control series documenting detailed headaches and other clinical characteristics could provide more information on the topic in the future.

#### 3.2.9. Imaging in RCVS and Temporal Relation of Symptomatology and Vasoconstriction

Imaging is of vital importance in RCVS to confirm vasoconstriction, exclude other causes of TCH, such as aneurysmal SAH or cerebral vasculitis, and monitor for potential complications [93]. The first imaging exam performed in a patient with TCH due to RCVS in the emergency department is usually non-contrast computed tomography (CT). The exam may be negative in more than half of the cases [80]. Digital subtraction angiography (DSA) has, by definition, close to 100% sensitivity for detecting peripheral vasoconstriction compared to CT angiography (CTA) and MRA, as it can assess vessel size to a diameter of 0.4 mm, compared with 0.7 mm in CTA. DSA does, however, have drawbacks, particularly the risks of an invasive vascular procedure and radiation exposure. Cerebrovascular complications have been described, including TIA, ischemic strokes, arterial dissection, or vessel perforation (TIA in 9% of the patients, and iatrogenic dissections in 0.14%) [90]. Complications of DSA specific to RCVS patients have not been clearly described. DSA tends to be reserved only for patients with negative findings of non-invasive techniques or for cases that also require intraarterial treatment [93]. The sensitivity of CTA or MRA has been reported to be around 80% for both techniques in the seminal case series by Ducros [5,93]. This number has been cited in the literature ever since, but no other sensitivity data have been adequately reported so far. Sensitivity analysis is complicated by the temporal progression of the condition. Nevertheless, MRA has been shown to be a good alternative to DSA, particularly since it enables concurrent detection of other pathology and easy monitoring of vasoconstriction progression.

Magnetic resonance (MR) is frequently performed in the setting of suspected RCVS to detect complications, such as intracerebral bleeding or ischemic stroke [80]. Consequently, concurrent MRA has been increasingly used to detect segmental vasoconstriction. It can detect medium- to large-sized arteries, giving a typical “string of bead” or “sausage on a string” appearance. Arterial spin labeling (ASL) is a perfusion MR technique, which enables cerebral blood flow (CBF) evaluation, without the need for gadolinium injection [80]. According to Kano et al., ASL can infer vasoconstriction in distal vessels “hidden” from MRA by detecting areas of hypoperfusion [94]. In this study, vasoconstriction reached its peak in the second week with subsequent gradual normalization. Centripetal progression of vasoconstriction from the distal arteries to the major cerebral arteries of the circle of Willis was observed in all patients [94].

Finally, transcranial doppler (TCD) might also be a useful tool in the identification of vasospasm in patients with probable RCVS. A study of 36 patients with RCVS who underwent TCD on an average of 16 (6–26) days after headache onset showed that 29 of them had mild or moderate vasoconstriction, which completely resolved on an average of 41 days after headache onset [95]. TCD has also been used to demonstrate that vasoconstriction increases gradually and peaks between 13 and 14 days after symptom onset [35]. Another small cohort study of 15 patients also demonstrated that TCD has potential as an initial non-invasive diagnostic method and particularly as a method for monitoring patients with RCVS [46]. TCD could be the best way to look for the peak of vasoconstriction in large-sized vessels as a non-invasive, easy, and relatively fast imaging examination given an adequate acoustic window.

As far as the timing of vasoconstriction is concerned in relation to symptom onset, our search of published observational studies demonstrated that visible vasoconstriction could occur on the same day as symptom onset, as has been reported by Shimoda et al. [56]. There are also reports of vasoconstriction being visible 1–3 days afterward [25,31], but it is most commonly evident some days into the disease course [18,37,44,46]. According to two reviews focusing on the neuroimaging evaluation of the syndrome, the peak of vasoconstriction is around 16 days after clinical onset, and it resolves within 3 months [80,81]. The percentage of patients with visible vasoconstriction at onset could be underestimated due to a delay in imaging acquisition. On the other hand, there are reports of negative imaging examinations, even when they are performed early in the course of the disease [3,96]. It remains unclear whether this can be attributed to a low sensitivity of imaging methods in detecting vasoconstriction of small, distal vessels that have not propagated yet proximally, as some theories suggest, or to a true absence of vasoconstriction at that time point.

Ducros et.al theorized that the pathological process first includes distal arteries and then progresses toward the branches of the circle of Willis [5]. Vasoconstriction of small distal arteries is undetectable on angiography and may stimulate trigeminal leptomeningeal afferents to induce thunderclap headaches [97]. Due to the difficulty in detecting vasoconstriction in very distal branches, it may be impossible to detect it during the first week of the disease [93]. Chen et al. thoroughly investigated the evolution of vasoconstriction in patients with RCVS in a study of 77 patients, demonstrated that diffuse vasoconstriction peaks on day 16.2 ± 9.4 (range, 2–51) and found that different vessels can be affected at different times in the disease course [6]. In 2016, Shimoda et al. demonstrated that centripetal propagation of vasoconstriction can be depicted on magnetic resonance angiography (MRA) when performed at the time of remission of TCH [56]. Two years later, the same group performed a retrospective, case–control study and analyzed data from 48 patients with RCVS who underwent an MRI within 72 h of RCVS onset and 48 h of TCH remission. Half of these patients showed centripetal propagation of vasoconstriction (CPV). These patients had an increased risk of brain lesions and a longer interval between the first and last TCH. By repeating MRA in the period between symptom onset and TCH remission, assessing CPV can be useful for the diagnosis of RCVS. The study hypothesized that CPV gradually progresses after the onset of RCVS, peaks at the time of TCH remission, and does not progress further afterward [57]. Fukaguchi et al. also propose performing clinical and imaging follow-ups for at least 2 weeks to exclude the syndrome [31].

### 3.3. RCVS Headaches in Children vs. Adults

RCVS commonly affects patients in the middle decades of life, though all age groups, including children, can be affected. However, there are not many published data on the clinical characteristics of the syndrome in children. Clinical diagnosis of RCVS in this age group may be more challenging due to the higher prevalence of inflammatory vasculopathy, differences in presentation, and difficulties in conducting diagnostic and confirmatory imaging in children, as well as in history taking. The latter makes acquiring information on headache characteristics and intensity particularly difficult. In most observational studies discovered by this literature search, patients under 18 years old had not been included or were very few in number. Published case reports have been expertly summarized in a recent review [98]. Our search resulted in a few more reported cases [78,99,100,101,102,103,104,105,106]. Compared to adults, male patients are more common in children, particularly adolescents, and RCVS occurs more commonly in the setting of some underlying diseases, such as hematologic disorders. Regarding headache characteristics, TCH or severe headaches remain the most common. Common triggers in children may be contact with water [107,108,109], exertion [99,110,111,112], and various medications [78,113,114,115].

### 3.4. RCVS and COVID-19

Compared to other recent reviews [72,89], our search resulted in more published case reports and series of RCVS in people with COVID-19 [105,116,117,118,119,120,121,122]. The most convincing argument on the association of COVID-19 with RCVS remains a case series published in 2021 [116]. Nine patients had been diagnosed with COVID-19 in the past 30 days, while one patient was diagnosed 45 days before the diagnosis of vasospasm. However, that study as published has numerous limitations, primarily inadequate reporting of the recruitment strategy and diagnostic criteria used for RCVS. Furthermore, four out of ten patients had vasoconstriction in only one vessel, only two patients had a follow-up angiographic evaluation to confirm reversal or improvement of vasoconstriction, and only five of the patients had an RCVS2 score of 5 or more.

RCVS was reported in a single case of the analysis print of the Pfizer-BioNTech vaccine [123]. Two cases of RCVS after vaccination exist in the literature. In one case, symptom onset was 18 days after a repeat dose of the Moderna vaccine [124], and vasospasm of the right PCA was reversed seven days later after treatment with nimodipine. In the other published case [125], symptoms occurred after 12 h of the first Pfizer dose in a 30-year-old patient with a history of RCVS 3 years prior. However, in this case, no imaging whatsoever confirmed the recurrence of vasospasm, although the clinical presentation described resembles HaRCVS.

In the setting of COVID-19, vasoconstriction has been hypothesized to occur not due to the infection itself but rather the down-regulation of the ACE2-receptors by SARS-CoV2. This might lead to sympathetic hypertonia of the cerebral vessel walls and/or over-activation of the classic renin–angiotensin axis that leads to vasoconstriction, thus precipitating RCVS [117,118]. Nevertheless, with the extent of the vaccination program and the enormous numbers of infections and re-infections worldwide, one would expect this phenomenon to be more widespread and more widely reported in the literature during these past three years before one were to hypothesize a pure etiological connection between the two conditions. It is always possible, however, that many instances of this association have been missed if hemorrhagic or ischemic incidents were attributed to the widely accepted hypercoagulation phenomena associated with COVID-19 and vasospasm was never suspected and investigated angiographically.

## 4. Discussion

A thunderclap headache is traditionally the hallmark of RCVS since it was first described by Call and Fleming [2]. Our search demonstrates that many articles in the literature do not adhere strictly to widely accepted definitions of TCH. Studies with a strict definition show that the percentage of patients with the supposed hallmark symptom was, in fact, significantly lower. These findings come in contrast to previous reviews reporting 85–100% [72], 94–100% [69], and 95–100% [91] of patients having TCH. Foregoing the time-to-peak criterion, the majority of patients still experience acute, severe headaches that peak relatively quickly. It is not easy for a patient under excruciating pain to remember and report whether their headache reached maximal intensity within 1, 5, or 10 min, and in the setting of acute evaluation of a new headache, such differentiation does not seem practical, at least in the setting of RCVS differentiation. There is an obvious need to reevaluate the typical headache characteristics of RCVS using a more lenient term than TCH, including at least a wider time to peak intensity interval. Neurologists should be aware that the typical TCH may not emerge in over 25% of patients with RCVS according to some studies and should, therefore, proceed with angiographic evaluation when a patient describes a new-onset severe headache occurring in minutes or even a few hours, even in the absence of non-contrast CT findings, if the rest of the presentation is compatible.

Detailed characteristics of the typical headache associated with RCVS, such as duration, quantitative intensity, and accompanying symptoms, seem to be poorly reported in the large studies found with our search compared to publications on other primary and secondary headaches. Most information still relies on the early descriptions of the syndrome and older cohort publications. This could be due to a large number of retrospective studies not finding the required information and the ease of use of an umbrella term of TCH, bypassing a more detailed description of the clinical presentation. Patients with RCVS often arrive at emergency departments with alarming symptoms, and gathering relevant information on the specific characteristics of the headache may take a second seat in favor of ruling out threatening diagnoses and treating complications, particularly if there are associated focal neurologic deficits, mental changes, or seizures. Retrospective reporting of headache characteristics after the event is often sought from the patients but is prone to recall bias. More prospective studies focusing on headache characteristics (onset-to-peak, type of pain, remission with analgetic medicine, maximum intensity, location of pain, etc.) are of vital importance in order to better define the clinical spectrum of this relatively newfound syndrome.

At the same time, there is an increase in published data on patients presenting atypical features, which can include gradual severe headaches, non-severe headaches, or no headaches at all. There is a high probability that patients with non-severe headaches or no headaches will seek medical care only if they present complications from RCVS. Since the main pathophysiological theory suggests that the vasoconstriction of small distal arteries may stimulate trigeminal leptomeningeal afferents to induce thunderclap headaches [97], it is logical to assume that patients with atypical headaches or no headaches at all could have alternate pathophysiological mechanisms in play, which do not result in the vasoconstriction of peripheral cerebral arteries or which, despite vasoconstriction, do not stimulate leptomeningeal afferents. Alternatively, the progression of pathology may stop for some reason, or these patients may be less sensitive to these pain-inducing mechanisms. This might also be reflected in the fact that patients with RCVS often have comorbidities with other headache disorders compared to the general population [126].

Concerning the main proposed pathophysiological basis of RCVS, Wolff et al. have suggested that there is a variety of possible underlying mechanisms to explain the change in the adrenergic vascular tone response, which starts distally and progresses proximally on cerebral arteries. These could include a genetic predisposition like the BDNF gene polymorphism, an imbalance of proangiogenic and antiangiogenic factors, or an endothelial dysfunction with oxidative stress. Some of these changes have also been elucidated in a study investigating circulating miRNA [127], implicating the miRNA-EDN1 feedback loop and impairment of cerebral autoregulation. The variety of clinical presentations with different types of headaches, accompanying symptoms, and other clinical characteristics that different patients manifest suggest that individual factors may come into play [70].

This literature search produced a large number of review articles pertaining, among others, to the differential diagnosis of the thunderclap headache, but also RCVS specifically. This number is disanalogous to the published primary research articles on the topic, which is a common problem in modern academic publishing. However, this particular review adds to the current body of knowledge in two ways. Firstly, it primarily identifies and presents publications of a higher level in the research pyramid to exact some of the conclusions, and secondly, it focuses on particular topics pertaining to the clinical diagnosis of the syndrome, such as headaches and the detailed characteristics associated with them. One disadvantage of our approach is that it foregoes case reports and small case series in the systematic approach. Articles such as these often prove useful in describing rare conditions and commonly contain important details on the presentation, timing of events, and diagnostic findings. However, we believe the literature has now matured enough to focus mainly on larger studies since acquiring information from isolated case reports and small series always has a ceiling and is prone to report bias. Perhaps a new systematic analysis of all case reports published, such as the recent one by Song et al. [77], but with a focus on headache characteristics similar to the Wolff et al. 2016 study [70], is warranted; although efforts should now focus in large, multi-national registries.

## 5. Conclusions and Future Directions

RCVS is a rare condition presenting with TCH occurring either spontaneously or in a broad range of clinical situations. It is an increasingly recognized condition due to modern radiological developments and a higher degree of suspicion. Most patients with RCVS recover completely, but in some cases, the outcome is unfavorable, leaving permanent disability and, in very few cases, it might be even fatal. Surprisingly, the description of its clinical presentation regarding headache characteristics and its temporal relationship to typical angiographic findings still relies mainly on retrospective, older cohort studies and case series. More contemporary large studies have not focused on these facets of presentation. Prospective studies using validated headache questionnaires in all patients with new-onset headaches comparing cohorts based on final diagnosis (RCVS, aSAH, first episode of a primary thunderclap, etc.) would give further insight into this syndrome and its clinical presentation. An international collaboration has recently been established to further elucidate RCVS (REVERCE) [128] and is expected to provide more concrete data on many facets of this fascinating syndrome.

## Figures and Tables

**Table 1:** Large series and cohort RCVS studies or other analytical studies with RCVS cohorts with information on headache characteristics.

	Type of Study, Population, and Aim	RCVS Cohort Size	TCH *n* (%)	TCH Definition Used	Baseline Headaches *n* (%)	Non-TCH Headaches *n* (%)	Non-TCH Characteristics	Accompanying Symptoms	Absence of Headaches *n* (%)
**Studies with general RCVS Cohorts ^1^**									
Ducros 2007 [3]	Prospective description of RCVS patients	67	66 (98.5)	HIS 2004	(73)	1 (1.5%)	Acute, severe, >1 min to reach peak	Nausea (57%), vomiting (38%), agitation (32%), photophobia (30%)	0
Chen 2008 [21]	Prospective analysis of TCD in RCVS cohort vs. controls	32	32 (100) (by design)	BACNS ICHD-2	17 (53)	NR	NR	Nausea (63%), photophobia (41%), vomiting (38%), phonophobia (34%)	0 (by design)
Chen 2010 [6]	Prospective analysis of MRA findings in RCVS	77	77 (100) (by design)	BACNS ICHD-2	NR	0 (by design)	NR	NR	0 (by design)
Singhal 2011 [7]	Retrospective analysis of MRA findings in RCVS	139	(85)	Severe, acute headache	NR	(10)	NR	NR	(5)
Choi 2018 [25]	Prospective description of a Korean RCVS cohort	104	90 (86.5)	Onset-to-peak <1 min	NR	11 (10.6)	Severe headache of gradual onset (peak from 2 to 120 min). Two wake-up	NR	3 (2.9)
Caria 2019 [18]	Retrospective description of an Italian RCVS population	102	69 (67.6)	Onset-to-peak <1 min	Yes, in most patients of mild severity	33 (32.4)	severe headaches of gradual onset, 14.7%. Throbbing headaches, 7.4%. Persistent headaches of variable severity, acute headaches peaking >1 min	Nausea and vomiting in 81.2% of TCH patients	0
Strunk 2022 [61]	Retrospective RCVS population, angiographic nimodipine test	13	8 (61.5)	NR	NR	5 (38.5)	NR	NR	0
Lange 2022 [42]	RCVS TCH/Non-TCH	345	281 (81.4)	Reaching a maximum intensity above 7/10 in <1 min	NR	55 (15.9) ^2^ 27 (7.8) ^3^	Excruciating reaching max <10 min *n* = 13, >1 h *n* = 2, unusual progressive *n* = 22, mild *n* = 2, wake-up *n* = 13	NR	9 (2.6)
**Studies with an RCVS sub-cohort ^1^**									
Neel 2012 [52]	Retrospective, RCVS vs. PACNS	13	10 (76.9)	“Similar to thunderclap”	NR	3 (23.1)	NR	NR	0
Cheng 2014 [23]	Prospective, patients with sudden headache	25	11 (45.8)	ICHD-2	11 (44)	14 (45.2)	Sudden headache	NR	5 (26%)
Wolff 2015 [67]	Prospective, young patients with AIS and RCVS	21	0	NR	11 (44%)	14 (74)	Unusual and severe	NR	0
Kim 2015 [37]	Retrospective, RCVS in TCH patients that did imaging	18	18 (100) (by design)	Sudden severe headaches that reached maximum intensity within minutes	All (unclear)	0 (by design)	NR	Nausea (7/18), ocular pain (2/18), vomiting (2/18), dizziness (1/18), phonophobia (1/18)	0 (by design)
Singhal 2016 [59]	Retrospective RCVS vs. PACNS	159	(89) ^1^	NR	NR	(7) ^1^	NR	NR	(3) ^1^
Graff-Radford 2016 [33]	Retrospective, convex SAH patients	26	21 (80.7)	NR	NR	2	NR	NR	3 (unclear)
de Boysson 2018 [28]	Retro-prospective, RCVS vs. PACNS	173	163 (94)	NR	NR	NR	NR	NR	NR
Smith 2021 [60]	Retrospective RCVS cohort with acute headaches	56	56 (100) (by design)	Any increase in headache intensity occurring within a 1 min timespan.	10 (17.8)	0 (by design)	Peak pain intensity as neither severe nor 8/10 or greater (in some episodes)	NE	0 (by design)
Lin 2021 [47]	Prospective cohort study, headache after sexual activity	174	151 (86.8)	Onset-to-peak <1 min	NR	NR	NR	Nausea 31%, vomiting 14.9%, photophobia 14.4%, phonophobia 27%	0 (by design)

^1^ Some of these studies come from the same research groups and probably share patients between cohorts. ^2^ At disease onset. ^3^ During entire disease course. AIS: acute ischemic stroke. MRA: magnetic resonance angiography. NR: not adequately reported. PACNS: primary angiitis of the central nervous system. RCVS: reversible cerebral vasoconstriction syndrome. SAH: subarachnoid hemorrhage. TCH: thunderclap headache.

## Data Availability

No datasets were analyzed or generated during the course of the current study. Results of the literature review process can be replicated by conducting the search as detailed in the methodology section.
